# Pin1 coordinates HDAC6 upregulation with cell migration in lung cancer cells

**DOI:** 10.7150/ijms.50097

**Published:** 2020-09-21

**Authors:** Hsiang-Hao Chuang, Jui-Feng Hsu, Hsu-Liang Chang, Pei-Hui Wang, Po-Ju Wei, Da-Wei Wu, Ming-Shyan Huang, Michael Hsiao, Chih-Jen Yang

**Affiliations:** 1 Division of Pulmonary and Critical Care Medicine, Department of Internal Medicine, Kaohsiung Medical University Hospital, Kaohsiung Medical University, Kaohsiung, Taiwan.; 2 Department of Internal Medicine, Kaohsiung Municipal Ta-Tung Hospital, Kaohsiung Medical University, Kaohsiung, Taiwan.; 3 Division of Hematology and Oncology, Department of Internal Medicine, Kaohsiung Medical University Hospital, Kaohsiung Medical University, Kaohsiung, Taiwan.; 4 Department of Internal Medicine, Kaohsiung Municipal Siaogang Hospital, Kaohsiung Medical University, Kaohsiung, Taiwan.; 5 Department of Internal Medicine, E-DA Cancer Hospital, Kaohsiung, Taiwan.; 6 School of Medicine, I-Shou University, Kaohsiung, Taiwan.; 7 Genomics Research Center, Academia Sinica, Taipei, Taiwan.; 8 Department of Respiratory Therapy, College of Medicine, Kaohsiung Medical University, Kaohsiung, Taiwan.; 9 School of Medicine, College of Medicine, Kaohsiung Medical University, Kaohsiung, Taiwan.

**Keywords:** HDAC6, Pin1, protein stability, migration

## Abstract

Histone deacetylase 6 (HDAC6) controls many cellular processes via its catalyzing deacetylation of downstream substrates or interacting with its partner proteins. Dysregulation of HDAC6 signaling links to many diseases. Our previous study has been reported peptidyl-prolyl cis/trans isomerase, and NIMA-interacting 1 (Pin1) involving in HDAC6-mediated cell motility. To gain insight into precisely coordination of HDAC6 and Pin1 in cell migration, shRNA-mediated gene silencing and ectopic expression were applied to manipulate protein expression level to evaluate relationship between HDAC6 and Pin1 expression. Quantitative RT-PCR and the cycloheximide (CHX) chase assay resulted in HDAC6 expression is correlated with Pin1 level in H1299 cells. It hints that the Pin1 increases HDAC6 expression through increased transcripts and posttranslational stabilization. Furthermore, wound healing assay and transwell invasion assay evidenced the contribution of Pin1 on cell motility in H1299 cells. Our data suggest that Pin1 acts as an important regulator to manage HDAC6 expression for cell motility in lung cancer cells.

## Introduction

Lung cancer is one of the deadliest malignancies worldwide 1. Though advanced therapeutic strategies were developed to treat lung cancer, the prognosis of lung cancer is still poor 2. Tumor metastases and drug resistance are the two major causes of cancer-associated morbidity and mortality 3, 4. Metastasis consists of serial processes 5. Briefly, spreading of tumor cells relies upon cell motility, which leads to invasion in neighboring connective tissue, to entry into blood or the lymphatic vessels (intravasation), to exit of tumor cells from vessels (extravasation), and to migration of tumor cells into distant tissues and expansion of the metastatic colonies 6, 7. Cell motility is the ability to direct and to move towards chemoattractant gradients (directional) for growth and survival 8. For tumor cells, the cell motility pathologically contributes to tumor cell survival and growth, as well as metastatic spreading 7, 9, 10.

Histone deacetylase 6 (HDAC6) is the unique member in the HDAC superfamily, which masters dynamically protein acetylation. HDAC6 is mainly retained in the cytoplasm, where it forms protein complex with noncovalent interaction with its partner proteins and modulates many important biological events. Enzymatically, the HDAC6 catalyzes the deacetylation of ɑ-tubulin, cortactin, heat shock protein 90, β-catenin and peroxiredoxins. This enzymatic modification achieves biological regulation on cellular events, such as cell motility, cilium assembly, cell adhesion, inflammation, angiogenesis, transcription and protein degradation 11. Dysregulation of HDAC6 is taken account into several pathological developments, such as cystic kidneys 12, neurodegenerative diseases 13 and tumorigenesis 14-16. Currently, HDAC6 is regarded as a prognostic marker for several cancers 17-20. At present, it is known that activation of estrogen or K-ras signaling cascade either upregulates HDAC6 expression for tumorigenesis 21, 22. It is characterized that microRNAs such as miR-22, miR-26a, miR-206, miR-221, miR-206 and miR-601, are able to downregulate HDAC6 expression 23-28. The downregulation of HDAC6 is elaborately regulated by the proteasome-dependent fashion 29. However, the whole picture of biological regulation on HDAC6 expression is not yet fully figured out.

The peptidyl-prolyl cis/trans isomerase, NIMA-interacting 1 (Pin1) isomerizes the phosphorylated peptidyl-prolyl peptide bonds to convert protein conformation 30-32. This configuration change as a molecular switch governs a wide variety of biological processes 33, 34. Pin1 regulates cyclin D1 expression through the catalytic action as a configuration switch, leading to the transcriptional upregulation and posttranslational stabilization 35-37. Consequently, this regulation increased the proliferation rate. On the contrary, loss of Pin1 in the mouse leads to cell-proliferative abnormalities resembling cyclin D1-null phenotypes 37. Elevation of Pin1 level markedly appears in human cancers 38. Therefore, it is regarded as a central modulator of tumor progression. Additionally, it has been suggested that Pin1 promotes drug resistance (chemotherapeutic agents, and tyrosine kinase inhibitors, TKIs) through an epithelial-mesenchymal transition (EMT) in lung cancer, breast cancer and cervical cancer, respectively 39-41. An increasing body of evidence shows that Pin1 serves as a poor prognostic marker for many cancers 38, 42-44. Apart from tumorigenesis, Pin1 acts a critical factor in the development of Alzheimer's disease 33, 45, 46. Of Pin1 functionalities, the protein stability, and functional interaction of Pin1 with its subtracts are managed through deacetylation of its K46 by HDAC6 47. Our previous study has been corroborated that HDAC6-Pin1 signaling cascade-mediated cell motility 48. It implies that a reciprocal interaction between the enzyme and substrate. However, the correlation between HDAC6 and Pin1 is largely unknown.

In the present study, we attempted to elucidate the relationship between HDAC6 and Pin1 in lung cancer. We observed that HDAC6 expression is correlated with Pin1 levels in various non-small lung cancer cells as well. And we also found that Pin1 is able to upregulate HDAC6 through increased transcripts and posttranslational stabilization. The expression levels of Pin1 and HDAC6 associate with cell motility.

## Methods

### Materials

Detailed information on the materials is listed in Supplementary Table S1.

### Cell culture

PC14 was obtained from Lee's laboratory at the National Cancer Center Hospital, Tokyo, Japan 49. CL1-0 and CL1-5 were obtained from Dr. Chu and colleagues at National Taiwan University, Taiwan, respectively 50. PC9 Iressa resistant cells were obtained from Dr. Yeh and colleagues at Taipei Medical University, Taiwan 51. Other lung cancer cell lines were purchased from the American Type Culture Collection (ATCC). The detail information about the cell lines were listed in Supplementary Table S1. The HEK293T and PC14 cells were cultured in Dulbecco's modified Eagle medium (DMEM) (Gibco, Grand Island, NY, USA). The other NSCLC cells were cultured in RPMI 1640 medium. The two media were supplemented with 10% fetal bovine serum (FBS) (Gibco, Grand Island, NY, USA), penicillin and streptomycin (100 U/mL). All cells were maintained at 37°C and 5% CO_2_ in humidified incubator.

### Western blot analysis

The cells were harvested and the lysates were prepared in 1× RIPA buffer containing protease inhibitors and phosphatase inhibitors. Protein concentrations were determined with a Bio-Rad DC protein assay kit. The 30 μg of protein lysates were resolved in a 7-15% SDS-polyacrylamide gel electrophoresis and then transferred onto a PVDF membrane. The protein bands were identified and probed with primary antibodies, and followed by horseradish peroxidase-conjugated secondary antibodies and displayed by enhanced chemiluminescence solution (GE Healthcare).

### Semi-quantitative RT-PCR

Total RNA was prepared from the H1299 cells overexpressing GFP or GFP-Pin1 using TRIzol Reagent (Invitrogen) according to the manufacturer's instructions. The cDNA was generated from 1 μg of purified total RNA from cells mentioned above using MMLV reverse transcriptase (Invitrogen). The synthesized cDNA samples were subjected to polymerase chain reaction (PCR) amplification and the cDNA yields were determined by the signals from the internal house-keeping genes glyceraldehydes 3-phosphate dehydrogenase (GAPDH) after amplification for 30 cycles. The primer sequences used in this study were listed in the Supplementary Table S1. The PCR products were subjected to electrophoresis on a 2% agarose gel with 1 μg/ml ethidium bromide. The images of DNA in the gel were captured by a UVP Bioimaging Systems EpiChemi3 Darkroom and analyzed using the ImageJ software.

### Real-time quantitative PCR

The isolation of total RNA and generation of cDNA were described above. The quantitative PCR was performed under the following conditions: 95°C for 2 min, followed by 40 cycles at 95°C for 15 sec and 65°C for 1 min. The real-time quantitative PCR was performed with the PowerUp SYBR Green Master Mix (Applied Biosystems, Carlsbad, CA, USA) using the 7900HT fast real-time PCR system (Applied Biosystems). The specific PCR primers used in this study were listed in Supplementary Table S1. The relative copy number was calculated using the threshold cycle (Ct) as calculated by the 7900HT fast real time PCR software. The target messenger gene (mRNA) expression levels were normalized with that of GAPDH and calculated by the 2^-ΔΔCt^ method.

### mRNA stability assay

The H1299 cells overexpressing GFP or GFP-Pin1 were treated with 2μM actinomycin D for 0, 6, or 12 hours and then were collected to isolate the total RNA. Generation of cDNA and the quantitative PCR were performed as described above.

### Wound healing assay

The cell migration of H1299 cells were measured by the scratch wound healing assay. Briefly, the cells (5 × 10^5^) were suspended in the complete media into 12-well plates and incubated at 37 °C and 5% CO_2_ in a humidified atmosphere overnight. The wound was generated by tip scratching and then transferred into an incubation chamber settled on an inverted microscope (Leica DMI6000 B) for time-lapse imaging.

### Transwell invasion assay

Cell invasion ability was determined by using Matrigel (CORNING) coated filter inserts (8-μm pore size) fitted into a 24-well plate (Merck Millipore, Billerica, MA, USA). Briefly, 200 μl of serum-free medium containing 2 × 10^4^ cells was inoculated onto the upper chamber of the transwell. 800 μl complete medium (supplemented with 10% FBS) was loaded onto the lower chamber and incubated for 18 hours at 37˚C. Then, the filter inserts were fixed with 4% formaldehyde and permeabilized by using 0.5% Triton X-100. After fixation, the cells were stained with 10% Giemsa solution. The filter membranes were washed twice with PBS, and the cells on the top surface of the filter were removed using cotton swabs. The cell number was counted under a fluorescence microscope (Nikon ECLIPSE Ti) at magnification × 100.

### Lentiviral production and infection

The shRNA lentiviral vectors were obtained from the National RNAi Core Facility, Academia Sinica, Taiwan. Production and infection of lentiviruses were performed by following the guidelines of the National RNAi Core Facility. Briefly, the lentivirus particles for all expression plasmids and shRNAs were prepared by co-transfection with the ΔR8.91 and pMD.G plasmids into HEK293T cells. The virus soup was collected, purified and introduced to cells to establish the stable clones.

### Statistical analysis

A two-tailed Student's *t*-test was used for statistical analyses. The data in this study were represented as the mean ±standard deviation of at least three independent experiments. *P< 0.05 and **P < 0.01 indicated significant differences among the experimental groups.

## Results

### Elevation of HDAC6 expression and Pin1 is coincident

**Figure 1:**
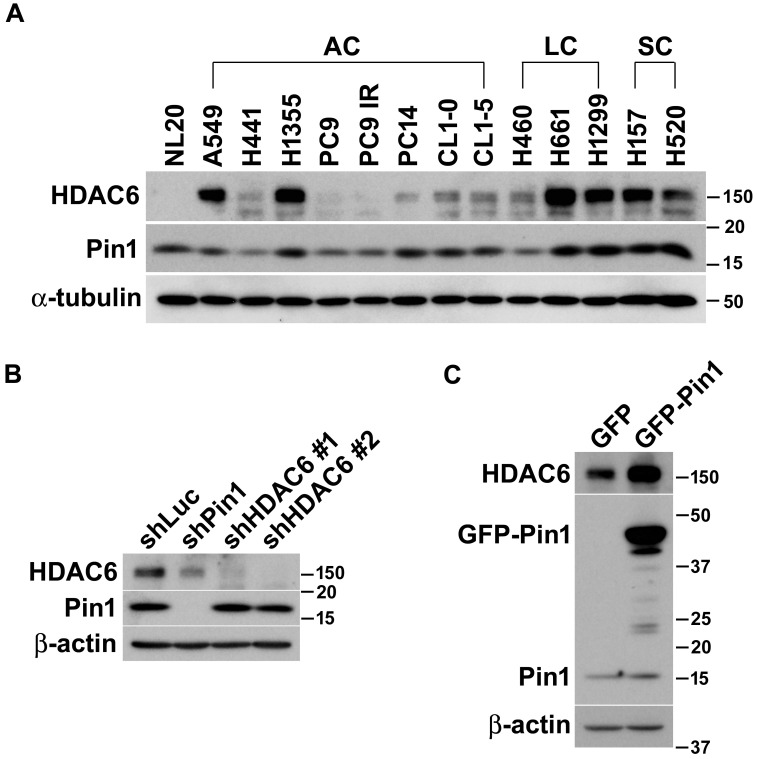
Pin1 influences HDAC6 expression in lung cancer cells. (**A**) The lung epithelial cells (NL20) and in 13 different non-small lung cancer cell lines were harvested and lysed in lysis buffer. The lysates were subjected to Western blot analysis. The expression of HDAC6 and Pin1 was analyzed by Western blot in NSCLC cell lines and ɑ-tubulin expression was used for loading control. AC, LC and SC indicated adenocarcinoma, large cell carcinoma, and squamous cell carcinoma, respectively. (**B**) H1299 cells harboring shRNA against luciferase, Pin1 and HDAC6, respectively, were lysed and subjected to Western blot analysis. The antibodies against HDAC6 (Santa Cruz Biotechnology, CA, USA), Pin1 (Santa Cruz Biotechnology, CA, USA) and beta-actin (Sigma-Aldrich, MO, USA) were used for this experiment to show the expression level, respectively. Western blot analysis was showed that low HDAC6 levels in the cells with shPin1 and higher levels in the cells with shLuc. (**C**) H1299 cells harboring overexpression of GFP or GFP-Pin1 were lysed and subjected to Western blot analysis. The protein levels were showed by the antibodies as indicated. The result was showed that increased HDAC6 levels in the cells harboring Pin1 overexpression.

In our previous study, we have been characterized that HDAC6 is one of Pin1 substrates and the involvement of Pin1 in HDAC6-mediated cell motility concerns with tumor metastasis in lung cancer cells 48. Otherwise, we also observed that the enzymatic activity and protein stability of Pin1 is modulated by HDAC6 47. To advance the functional and biochemical relationship of HDAC6 and Pin1 in lung cancer, we firstly quantified the expressions of HDAC6 and Pin1 in a variety of non-small lung cancer cell lines. Generally, high expression level of HDAC6 is present in large cell and squamous cell carcinomas of non‑small cell lung cancer (NSCLC). The HDAC6 is abundant in A549 and H1355 cells in lung adenocarcinoma, but its expression levels are relatively lower in normal lung epithelial NL20 cells. Interestingly, the change in Pin1 expression levels in lung cancer cells line is similar to HDAC6 expression patterns in those cells lines. Intriguingly, higher expression levels of HDAC6 and Pin1 are coincidently present in a variety of lung cancer cells (Figure 1A).

### Pin1 controls HDAC6 expression

It is elusive that the higher expression levels of HDAC6 and Pin1 in the variety of lung cancer cell lines are coincident case or attribute to their biochemical or functional relevance. To dissect the functional and biochemical relevance between HDAC6 and Pin1, desired shRNAs were used to deplete HDAC6 and Pin1expression, respectively. As noted in figure 1, Pin1 depletion dramatically resulted in lowering HDAC6 expression in H1299 cells (Figure 1B). Conversely, HDAC6 depletion led to tiny effect on Pin1 expression levels (Figure 1B). This was also observed in gefitinib-resistant PC9 and H1975 cells (Figure S1). It seemed that the Pin1 is the upstream regulator determining HDAC6 expression levels in NSCLC cells. Similarly, ectopic expression of Pin1 in H1299 cells can gives effect on HDAC6 expression levels. The GFP-Pin1 overexpression showed persistent HDAC6 upregulation (Figure 1C). In addition to H1299 cells, we proposed to investigate whether the Pin1 overexpression could cause higher HDAC6 expression levels in HEK293T, A549, and H661 cells. Experimentally, it appeared that GFP-Pin1 overexpression persisted higher HDAC6 expression levels in those cells (Figure S2). It hinted that the Pin1 might involve in HDAC6 expression in NSCLC cell lines.

### Pin1 gives rise of HDAC6 expression through elevating transcript level, and posttranslational stabilization

**Figure 2:**
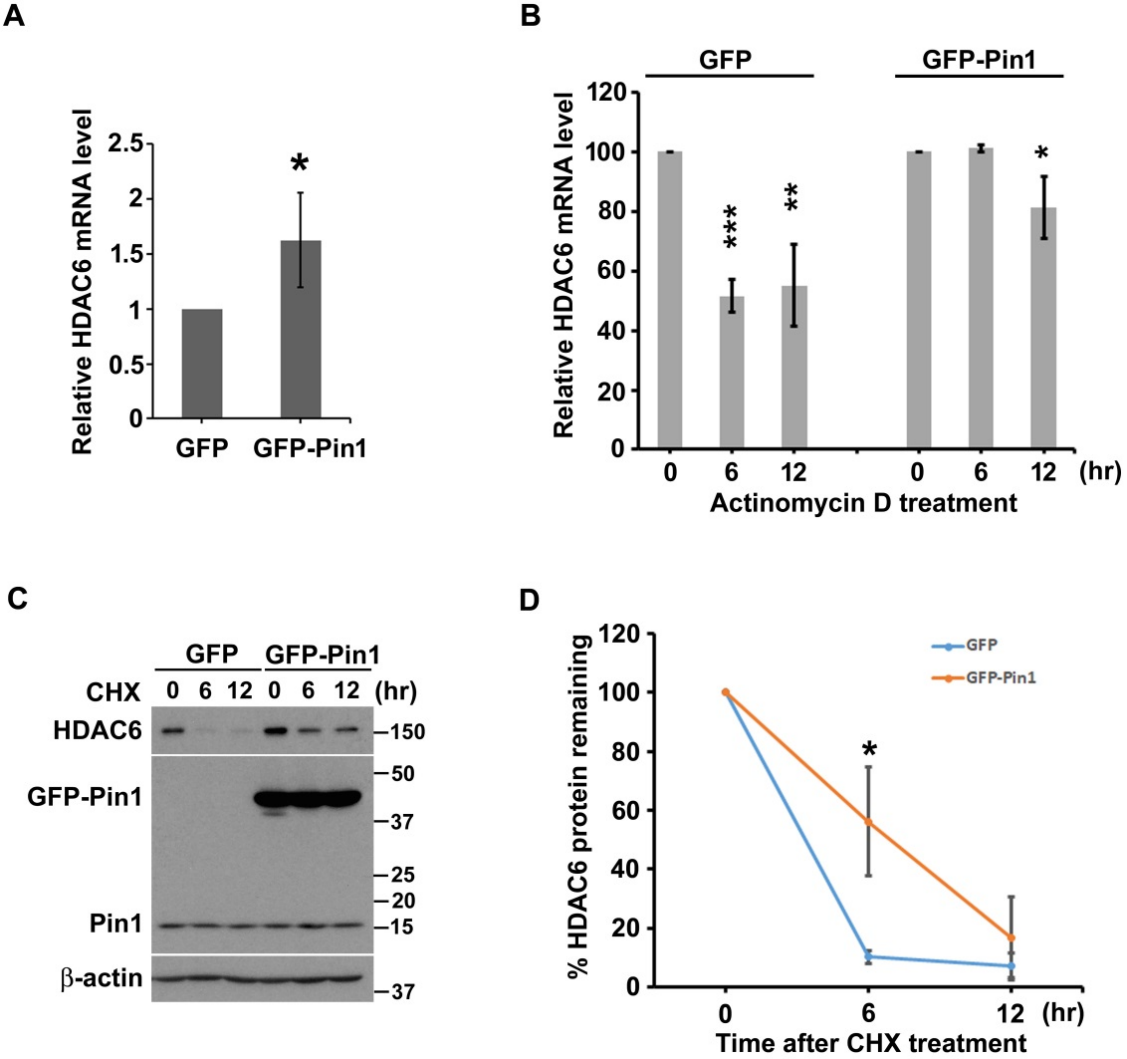
Pin1 overexpression increases the transcript level of and protein stability of HDAC6. (**A**) Total RNA in H1299 cells harboring overexpression of GFP or GFP-Pin1, was isolated and subjected to quantitative RT-PCR analysis. The threshold cycle (Ct) was calculated by the 7900HT fast real time PCR software. The expression levels of HDAC6 mRNA were normalized with that of GAPDH and calculated by the 2^-ΔΔCt^ method. The relative ratio of HDAC6/GAPDH to one in GFP group was plotted in a bar chart. Significant difference (P < 0.05) was showed between the GFP-Pin1 overexpressing group and GFP expressing one. Higher level of HDAC6 mRNA in H1299 cells with overexpressing GFP-Pin1 was found. The data represent the means ± SD from 4 separate experiments. (**B**) H1299 cells harboring overexpression of GFP or GFP-Pin1 were treated with 2μM actinomycin D for the indicated periods and then harvested for the quantitative RT-PCR analysis. The expression levels of HDAC6 mRNA were normalized with that of GAPDH and calculated by the 2^-ΔΔCt^ method. The relative ratio of HDAC6/GAPDH to untreated group was plotted in a bar chart. Significant difference (**P < 0.01 and ***P < 0.001) was showed between the actinomycin D treating group and untreated one. Highly stable HDAC6 mRNA in H1299 cells with overexpressing GFP-Pin1 was found. The data represent the means ± SD from 3 separate experiments. (**C**) H1299 cells harboring overexpression of GFP or GFP-Pin1 were treated with 100 μg/ml cycloheximide (CHX) for indicated periods. The treated cells were lysed and subjected to Western blot analysis with the antibodies indicated. lysed and subjected to Western blot analysis. The protein levels were showed by the antibodies as indicated. The result was showed that increased HDAC6 levels in the cells harboring Pin1 overexpression. (**D**) The relative ratios of HDAC6/β-actin normalized to one at t = 0 time point were quantified. The band intensities of HDAC6/β-actin were analyzed using the ImageJ software. The ratio of HDAC6/β-actin to untreated group was plotted in a bar chart. Significant difference (P < 0.01) was showed between the GFP-Pin1 overexpressing group and GFP expressing one. Highly stable HDAC6 protein in H1299 cells with overexpressing GFP-Pin1 was found. The data represent the means ± SD from 3 separate experiments.

As mentioned above that Pin1 gave effect on HDAC6 expression in the H1299 cells. In order to advance in understanding on how the Pin1 biochemically and functionally adjusts HDAC6 expression, we investigated the effect of Pin1 on transcript level or posttranslational stability of HDAC6. Experimentally, the semi-quantitative and quantitative RT-PCR were employed to quantify HDAC6 transcript levels under the background of Pin1 overexpression. The semi-quantitative RT-PCR data showed that HDAC6 mRNA expression levels are higher in the cells with GFP-Pin1 overexpression, compared to cells with GFP expression (Figure S3A). The mRNA amounts were statistically analyzed and plotted in the bar chart (Fig. S3B) (p≈0.002). Similarly, the HDAC6 mRNA expression is more in the cells with GFP-Pin1 overexpression than the cells harboring GFP as analysis achieved by quantitative RT-PCR (Fig. 2A) (p≈0.015). Those results indicate that Pin1 overexpression significantly associated with higher HDAC6 expression. Following, we attempt to understand what way the Pin1 sustain higher HDAC6 expression level in lung cancer cells. As the Pin1 is an enzyme involved in parathyroid hormone mRNA stability 52, the regulation of Pin1 on HDAC6 mRNA stability possibly occurs, the Pin1 overexpression could confer higher HDAC6 protein levels in the cells. Experimentally, the actinomycin D administration was used to estimate the mRNA stability. It appeared that more HDAC6 mRNA accumulated in the cells with GFP-Pin1 overexpression after actinomycin D exposure (Figure 2B).

Beyond higher HDAC6 transcript-levels, we also evaluate the effect of Pin1 on HDAC6 protein stability. Not only HDAC6 mRNA, the Pin1 overexpression can also promote HDAC6 protein stability as the cycloheximide (CHX) chase assay was evaluated. Our data shows that amount of HDAC6 protein was decreased with increment time of cycloheximide administration in the cells harboring GFP or GFP-Pin1 either (Figure 2C). The protein bands were digitalized with ImageJ software, and those values were statistically plotted in a line graph (Figure 2D). HDAC6 remaining rate fell to less 20% post CHX administration for 6 hours in cells with GFP expression. However, HDAC6 remaining percentage sustained more than 50% post CHX treatment for 6 hours in the cells with the GFP-Pin1 overexpression (Figure 2D). It indicated that the Pin1 overexpression could promote the protein stability of HDAC6. These results showed that the Pin1 can upregulate HDAC6 expression. The manners that Pin1 elevated HDAC6 expression levels not only increase HDAC6 mRNA levels but also give rise to HDAC6 protein stability.

### Pin1 promotes cell motility through upregulation of HDAC6

**Figure 3:**
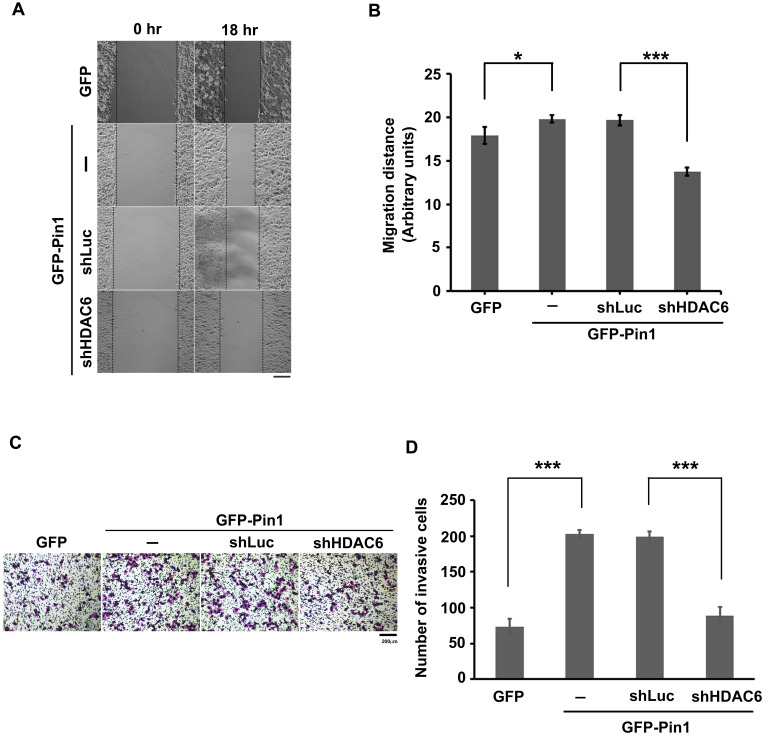
Pin1 promotes cell motility. (**A**) H1299 cells expressing GFP or GFP-Pin1, or harboring HDAC6 knockdown but containing GFP-Pin1 overexpression grew fully in wells. The wound was generated by tip scratching and cell migration was monitored by microscopy. The distance of migration was scored. (Scale bar, 200 µm) (**B**) The results of migration distances are shown as averages ± SD from three independent experiments compared to the GFP expressing group or the containing shRNA against luciferase group. *P < 0.05 and ***P < 0.01 based on Student's t-test. (**C**) The cell invasion activity of H1299 cells expressing GFP or GFP-Pin1 or harboring HDAC6 knockdown in the meanwhile containing GFP-Pin1 overexpression was measured by using Matrigel-coated transwell inserts followed by Giemsa staining. (Scale bar, 200 µm). Briefly, 2 × 10^4^ cells suspended in serum-free medium was inoculated onto the upper chamber of the transwell and the complete medium supplemented with 10% FBS was loaded onto the lower chamber. After incubation for 18 hours at 37ºC, the invasive cells were harvested for analysis. (**D**) The numbers of invasive cells by Giemsa staining were counted under a fluorescence microscope at magnification × 100. We randomly selected five fields to count the number of invasive cells. The data represent the means ±SD from 3 separate experiments. ***P < 0.001 based on Student's t-test.

**Figure 4:**
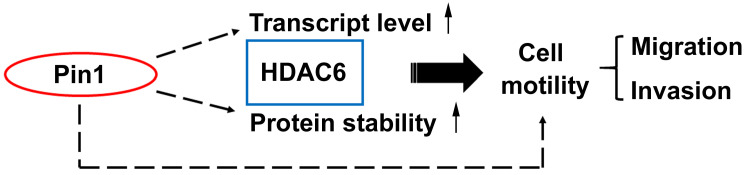
Pin1 enhances HDAC6 expression and promotes cell motility. A proposed mechanism for Pin1 promotes HDAC6 expression and cell motility. Pin1 overexpression increases HDAC6 expression through upregulation of HDAC6 transcript level and elevated posttranslational protein stability of HDAC6. In the meanwhile, Pin1 promotes cell motility partially through HDAC6 upregulation.

As described above, the Pin1 was able to increase HDAC6 mRNA amount and to stabilize protein posttranslationally. It is known that HDAC6 is an enzyme responsible for protein deacetylation. When its substrates, such as ɑ-tubulin and cortactin, are deacetylated, the microtubules get dynamics and microfilament reassembly is active, respectively. As consequence, the cytoskeletons dynamics drives the cells motility 53-55. To gain insight into involvement of Pin1 overexpression regulation on cell motility, the wound-healing assay was settled in this study (Figure 3A). The distance moving is evaluated and the values were plotted in a bar chart (Figure 3B). The distance moving in the cells with GFP-Pin1 overexpression slightly increased, compared to the cell with GFP overexpression (p≈0.033). In the meanwhile, HDAC6 knockdown (Figure S4) abolishes Pin1 overexpression-mediated cell migration (Figure 3A and 3B). To determine the invasion activity, the transwell inserts coated with matrigel were applied. We found that H1299 cells with ectopically expressing GFP-Pin1 have more invasive activity than the cells bearing GFP (Figure 3C). The numbers of invasive cells were counted and plotted in a bar chart (Figure 3D). It showed that the cells with Pin1 overexpression can dramatically gain invasive activity, compared to the cells with GFP (Figure 3D). At the same time, the cells with HDAC6 depletion impaired in invasive ability, no matter what the Pin1 overexpression-induced cell invasion (Figure 3C and 3D). In the present study, we demonstrated that Pin1 regulates HDAC6 expression. The regulation of Pin1 on HDAC6 expression improves cell motility in lung cancer cells (Figure 4).

## Discussion

Of systematic protein regulations in eukaryotes, post-translational modification (PTM) is the key mechanism managing enzyme activity, protein compartmentalization, proteins assembled into complexes, and protein degradation. For example, protein phosphorylation that modulating catalytic activity or organizing protein complex in given biological processes is an important regulatory mechanism in eukaryotic cells. Along with protein phosphorylation, Pin1-mediated phosphorylation-dependent protein isomerization has been identified as a crucial regulator in cellular signaling and diseases 33, 34. Beside phosphorylation, lysine acetylation is also a PTM on a variety of proteins as regulatory way to manage diverse biological processes 56. HDAC6 is an enzyme capable of protein deacetylation.

Dysregulation of HDAC6-mediated signaling commits the pathological progresses, such as cystic kidneys 12, neurological diseases 13 and cancers 14-16. As the HDAC6 involved in diverse pathological development, it was speculated that the HDAC6 could be a target to prevent pathological progression 15. Particularly, HDAC6 derived regulation mechanism remains incompletely understood. In the present study, we found that Pin1 is able to increase HDAC6 protein level (Figure 1C). Pin1 catalyzes the *cis*/*trans* isomerization of peptidyl-prolyl peptide bonds that act protein conformation change. This conformational conversion might advance a protein in its stability. In the present study, overexpression of Pin1 renders HDAC6 to a stable structure (Figure 2C and 2D). In the stereotactic regulation, changes in protein conformation could affect its transcription activity of a transcription factor or ability of RNA binding proteins either. Ectopic expression wild-type Pin1 increases HDAC6 transcript levels (Figure 2A and Figure S3). It also stabilizes HDAC6 mRNA (Figure 2B). However, we didn't know whether regulation of Pin1 on activity of transcriptional factors for HDAC6 expression or impairment in microRNA biogenesis by mediating conformation change of Exportin-5 (XPO5) which is actively involved in pre-miRNA nuclear export for function processing is the way to adjust HDAC6 levels in cells 57. The HDAC6 expression level can be regulated by microRNAs such as miR-22, miR-26a, miR-206, miR-221, miR-206 and miR-601. It needs to be fully investigated how the Pin1 acts in regulation of HDAC6 transcription.

Besides estrogen signaling, Ras signaling can also upregulate HDAC6 expression 21, 22. In this context, E2F is an effector mediating Ras and Neu signaling engaged with Pin1 expression 58. Ras signaling mediated expression of HDAC6 and Pin1. In the present study, we show that HDAC6 expression is correlated with Pin1 expression level in NSCLC cell lines (Figure 1A). Beside upregulation of HDAC6 and Pin1, the NSCLC cells that also bear Ras activation or constitutive active Ras. The HDAC6 is required for oncogenic Ras and Neu-induced transformation 16. In this oncogenic context, the HDAC6 and Pin1 expression is regarded to involve in tumor progression and poor prognosis. We evaluated the relevance of higher HDAC6 and higher Pin1 expressions and the survival rate using Kaplan-Meier Plotter database 59. High expression of HDAC6 associated with poor outcome in lung adenocarcinoma. However, the survival rate was not associated with HDAC6 in both squamous cell carcinoma and total lung cancers (data not shown). Based on the online database, Pin1 expression seems not associated with survival rate in lung cancers (data not shown). However, Tan et al., showed that high expression of Pin1 was correlated with poor survival and high expression of Pin1 was considering an prognostic factor for lung cancer 43. In conclusion, High expression levels of HDAC6 and Pin1 were found to be associated with poor outcome in lung cancer.

Advancing in involvement of HDAC6 expression in biological events, the Pin1 overexpression correlated to HDAC6 expression for cell motility was also evaluated. Pin1 overexpression increased cell migration in lung cancer cells. It is consistent to Wang's study in hepatoma cells 60. In this present study, we found that HDAC6 depletion impaired cell migration in the cell with Pin1 overexpression that can moderately activate cell invasion (Figure 3). The biological function of Pin1 is catalyzing protein isomerization and switching the protein properties. Pin1 overexpression increased the Gli1 protein level, a regulator of the EMT, by increasing the protein stability of Gli1, and promotes EMT and tumor cell motility 60. It is also found that Pin1 induced Akt-mediated GSK-3β inactivation and increased the level of Snail via transcriptional activation and protein stability, and finally caused EMT 41. Furthermore, isomerization of the protein tyrosine phosphatase (PTP)-PEST by Pin1 facilitates the interaction between PTP-PEST, and FAK and its dephosphorylation at Y397, which promotes cell migration, invasion, and metastasis 61. The Pin1 has been found to control cell migration through promoting EMT and Wnt/β-catenin signaling 40, 41, 62, 63. Furthermore, HDAC6 is required for Wnt/β-catenin signaling 64-66. According to these studies and our result (Figure 3), we inferred that existence of partial regulation on cell motility is a HDAC6-independent fashion. Our previous study and Dr. Penela's report had been found that interaction of HDAC6 and Pin1 is not following the rule of recognizing pSer/Thr-Pro motif in HDAC6 47, 48. The interaction of Pin1 and HDAC6 attributed to the conformational change of HDAC6 or assemblage of HDAC6-Pin1 complexes. Thus, this study suggested that anti-Pin1 is promising strategies to downregulate HDAC6 expression and can be considered an anti-metastasis in lung cancers.

## Supplementary Material

Supplementary figures and tables.

## Abbreviations

HDAC: histone deacetylase
NSCLC: non-small cell lung cancer
Pin1: peptidylprolyl cis/trans isomerase NIMA-interacting 1
PTM: posttranslational modification
EMT: epithelial-mesenchymal transition
RT-PCR: reverse transcription-polymerase chain reaction
GFP: green fluorescent protein
IR: Iressa resistant
CHX: cycloheximide
AC: adenocarcinoma
LC: large cell carcinoma
SC: squamous cell carcinoma
