# Supplementary material for: Pin1 coordinates HDAC6 upregulation with cell migration in lung cancer cells
Source: Int J Med Sci. 2020 Sep 21;17(17):2635–43. doi: 10.7150/ijms.50097 (PMC7645340; doi:10.7150/ijms.50097)
Supplement: Supplementary file 1 — Supplementary figures and tables. [file ijmsv17p2635s1.pdf]

Supplementary Material

Pin1 Coordinates HDAC6 Upregulation with Cell Migration in Lung Cancer Cells

Hsiang-Hao Chuang, Jui-Feng Hsu, Hsu-Liang Chang, Pei-Hui Wang, Po-Ju Wei, Da-Wei Wu, Ming-Shyan Huang, Michael Hsiao*, and Chih-Jen Yang*

*** Correspondence:**

Michael Hsiao: mhsiao@gate.sinica.edu.tw

Chih-Jen Yang: chjeya@cc.kmu.edu.tw

1. Supplementary Figures and Table

1.1 Supplementary Table

Supplementary Table S1

RAGENT or RESOURCE	SOURCE	IDENTIFIER	WORKING STATUS
**Antibodies**			
Anti-alpha Tubulin	SIGMA	Cat# SAB3501071	10k dilution
Anti-beta-Actin	SIGMA	Cat# SI-A5441-.2 ml	5k dilution
Anti-GFP	GeneTex	Cat# GTX113617	10k dilution
Anti-human HDAC6 Antibody (D-11)	Santa Cruz	Cat# sc-28386	500 dilution
Anti-human Pin1 (G-8)	Santa Cruz	Cat# sc-46660	1k dilution
**Cell lines**			
A549	ATCC	ATCC® CCL-185	RPMI 1640 + 10%FBS
CL1-0	Dr. Cheng-Wen Wu, Academia Sinica, Taiwan	N/A	RPMI 1640 + 10%FBS
CL1-5	Dr. Cheng-Wen Wu, Academia Sinica, Taiwan	N/A	RPMI 1640 + 10%FBS
H157	ATCC	ATCC® CRL-5802	RPMI 1640 + 10%FBS
H441	ATCC	ATCC® HTB-174	RPMI 1640 + 10%FBS
H460	ATCC	ATCC® HTB-177	RPMI 1640 + 10%FBS
H520	ATCC	ATCC® HTB-182	RPMI 1640 + 10%FBS
H661	ATCC	ATCC® HTB-183	RPMI 1640 + 10%FBS
H1299	ATCC	ATCC® CRL-5803	RPMI 1640 + 10%FBS
H1975	ATCC	ATCC® CRL-5908	RPMI 1640 + 10%FBS
H1355	ATCC	ATCC® CRL-5865	RPMI 1640 + 10%FBS
HEK293T	ATCC	ATCC® CRL-3216	DMEM + 10%FBS
PC9	Dr. TH Alexander Wu, Taipei Medical University, Taiwan		RPMI 1640 + 10%FBS
PC9 IR (Iressa resistant)	Dr. TH Alexander Wu, Taipei Medical University, Taiwan	N/A	RPMI 1640 + 10%FBS
PC14	Dr. Yung-Chie Lee, National Taiwan University, Taiwan	N/A	RPMI 1640 + 10%FBS
**Chemicals, Enzymes and Materials**			
Actinomycin D	SIGMA	Cat# A1410-2MG	2 μM
Dulbecco's Modified Eagle Medium	Gibco	Cat# 12100-061	
Fetal bovine serum	Gibco	Cat# 10437-028	
G418 (Geneticin)	Thermo Fisher	Cat# 10131035	800 μg/mL
GIEMSA	Merck Millipore	Cat# 1.09204.0100	
Lipofectamine 2000	Invitrogen	Cat# 11668019	
Matrigel	CORNING	Cat# 354234	
Millicell® Cell Culture Inserts	Merck Millipore	Cat# MCEP24H48	
MMLV Reverse transcriptase	Invitrogen	Cat# 28025013	

PowerUp SYBR Green Master Mix	Applied Biosystems	Cat# A25741	
Puromycin	InvivoGen	Cat# ant-pr-1	2 μg/mL
RPMI medium1640	Gibco	Cat# 31800-089	
TRIzol reagent	Invitrogen	Cat# 15596026	
**Recombinant DNA**			
pEGFP-C1	Clontech	CAt# 6084-1	
pEGFP-Pin1 WT	Dr. Kun Ping Lu, Harvard Medical School	N/A	
pCMVdeltaR8.91	RNAi Core Facility, Academia Sinica		
pMD.G	RNAi Core Facility, Academia Sinica		
Luciferase shRNA	RNAi Core Facility, Academia Sinica		
HDAC6 shRNA #1	RNAi Core Facility, Academia Sinica	TRCN0000004839	
HDAC6 shRNA #2	RNAi Core Facility, Academia Sinica	TRCN0000314910	
PIN1 shRNA	RNAi Core Facility, Academia Sinica	TRCN0000010577	
**Primers for PCR**	**Sequences**		
HDAC6 forward	AAGAAGACCTAATCGTGGGACT		
HDAC6 reverse	GCTGTGAACCAACATCAGCTC		
GAPDH-forward	TCTCCTCTGACTTCAACAGCGAC		
GAPDH-reverse	CCCTGTTGCTGTAGCCAAATTC		
**Other**			
Leica DMI6000 B microscope	Leica	N/A	
Nikon ECLIPSE Ti microscope	Nikon	N/A	
UVP Bioimaging Systems EpiChemi3 Darkroom	UVP	N/A	
7900HT Fast Real-Time PCR System	Applied Biosystems	N/A	

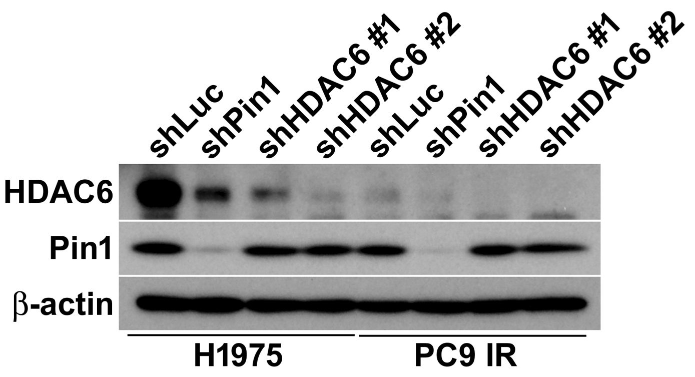

Figure S1. Downregulation of Pin1 reduces HDAC6 expression. H1975 and PC9 Iressa resistant cells harboring shRNAs against luciferase, Pin1 and HDAC6, respectively, were lysed and subjected to Western blot analysis. Western blot analysis was showed that low HDAC6 levels in the cells with shPin1 and higher levels in the cells with shLuc.

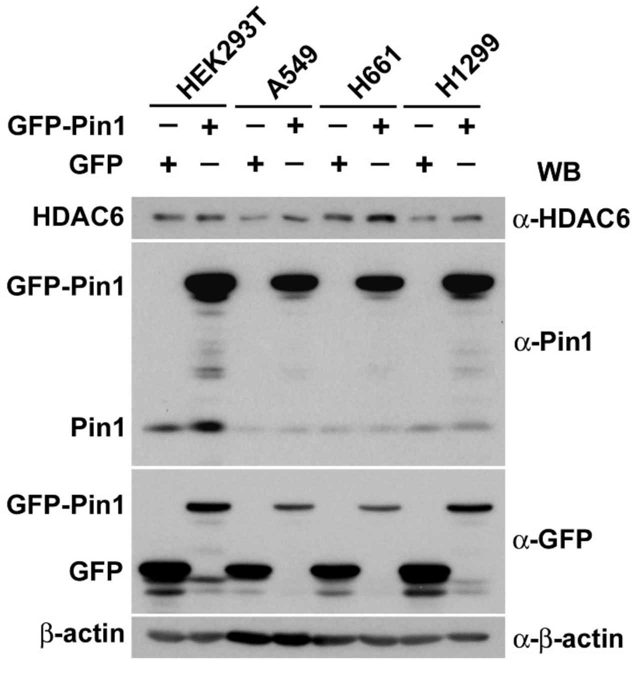

Figure S2. Overexpression of Pin1 increases HDAC6 expression. HEK293T, A549, H661, and H1299 cells were transfected with plasmid DNA of GFP or GFP-Pin1. 48 hours after transfection, the transfected cells were lysed and subjected to Western blot analysis. The antibodies against HDAC6, Pin1, GFP and beta-actin were used to show the protein expression level, respectively. The result was showed that increased HDAC6 levels in the cells harboring Pin1 overexpression.

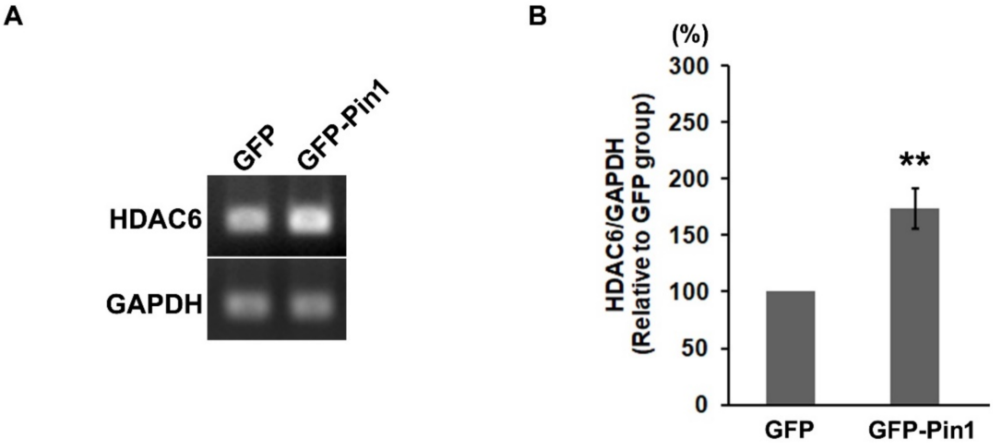

Figure S3. Overexpression of Pin1 increases HDAC6 transcript level. (A) Total RNA in H1299 cells harboring overexpression of GFP or GFP-Pin1, was isolated and subjected to semi-quantitative RT-PCR analysis. The GAPDH expression level was used for loading control. (B) The relative ratio of HDAC6/GAPDH to one in GFP group was plotted in a bar chart. Significant difference ($P < 0.01$) was showed between the GFP-Pin1 overexpressing group and GFP expressing one. Higher level of HDAC6 mRNA in H1299 cells with overexpressing GFP-Pin1 was found. The data represent the means ±SD from 3 separate experiments. **$P < 0.01$ based on Student's t-test.

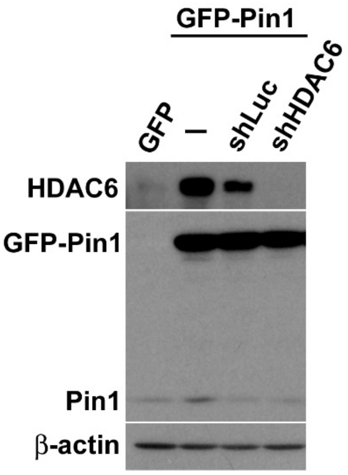

Figure S4. Characterization of HDAC6 depletion in H1299 cells expressing GFP-Pin1. H1299 cells expressing GFP or GFP-Pin1, or harboring HDAC6 knockdown but containing GFP-Pin1 overexpression were harvested and harvested and lysed in lysis buffer. The lysates were subjected to Western blot analysis. The

expression of HDAC6 and Pin1 was analyzed by Western blot and β-actin expression was used for loading control.
